# Villain of Molecular Biology: Why are we not reproducible in research?

**DOI:** 10.12688/f1000research.6854.1

**Published:** 2015-07-30

**Authors:** Vikash Bhardwaj

**Affiliations:** 1 Molecular Biology and Genetics Domain, Lovely Professional University, Punjab, India

**Keywords:** Reproducibility, Irreproducibility, Molecular biology, DNA, Research

## Abstract

Worldwide, there is an issue of ****irreproducibility in life science research. In the USA alone $28 billion per year spent on preclinical research is not reproducible. Within this opinion article, I provide a brief historical account of the discovery of the Watson-Crick DNA model and introduce another neglected model of DNA. This negligence may be one of the fundamental reasons behind irreproducibility in molecular biology research.

## Introduction

Every year billions of dollars are invested in research worldwide to find solutions for deadly diseases like cancer, AIDS, TB etc. Much research is now focused on DNA and everyone is trying to understand what is happening within DNA at the molecular level. Whenever I ask my students within a molecular biology class, “Who discovered DNA?” many of my students give a very quick response “Watson and Crick”. It reminds me of a statement made by L. Pray that “Many people believe that American biologist James Watson and English physicist Francis Crick discovered DNA in the 1950s” ^1^. Then a few students respond, “No sir” Watson and Crick uncovered the structure of DNA. But still it remains a question to my students: who discovered DNA? When there is no answer, I start explaining history of DNA discovery. I remind them about Friedrich Miescher who isolated DNA for the first time in 1869 and how Watson and Crick deciphered the structure of DNA in 1953. It took almost 85 years to discover the molecular details of DNA structure. Friedrich Miescher was a brilliant scientist but he suffered when his findings were not published immediately and his boss published his results after repeating his experiments on his own. It took almost two years to get his results published. Even Miescher had the opinion that the new substance (“nuclein”) could be thought of as genetic material but various established theories stopped his journey. Many scientists at that time were of the opinion that some proteins could be genetic material and they were busy finding novel proteins which could act as genetic material. Friedrich Miescher died in 1895 without getting credit for his discovery ^2^. Later in 1919, the “Tetra nucleotide hypothesis” proposed by Levene become an obstacle in DNA structure discovery as he proposed DNA as an inert molecule having four nucleotides repeatedly arranged. Levene was also a brilliant scientist and had published more than 700 papers and many scientists followed his opinions ^3, 4^. In the coming years scientists were not interested in doing work on DNA until 1928 when Griffith gave some evidence that it is not the proteins which acts as a genetic material ^5^. His experimental findings were worked out further in 1944 by Avery *et al.* and they clearly demonstrated that it is DNA which acts as a transforming agent but still they faced opposition. Still, many scientists were slow to accept this clear proof that DNA, not protein, is the genetic molecule ^6, 7^. From 1950 onwards Chargaff used to meet and discuss with scientists that he had different results showing that DNA cannot be an inert molecule thus clearly rejecting the “Tetra nucleotide hypothesis”. But his views were not given much importance ( http://www.dnai.org) ^8^. In 1952, Hershey and Chase did a classical experiment which proved without doubt that DNA is genetic material ^9^. It created interest in others to solve out molecular details of DNA. In 1953 Linus Pauling (one of the famous Nobel laureates) proposed a triple helical model of DNA in which they proposed phosphate groups are inside while nitrogenous bases are outside ^10^. Really Pauling’s authority in science might have become another obstacle in the discovery of the correct DNA model, if Watson and Crick had not realized that phosphate groups cannot be inside, as this would destabilize DNA due to high negative charge. Watson, Crick and Wilkinson proposed the double helical structure of DNA based on work done by Rosalin Franklin ^11– 13^. Linus Pauling himself visited Watson and Crick and was convinced about their proposed model ( https://paulingblog.wordpress.com/2009/04/30/the-watson-and-crick-structure-of-dna/). Watson, Crick and Wilkinson received the Nobel Prize in 1963 for solving the structure of DNA. Yes, truly their findings have changed molecular biology research worldwide. Many scientists started developing new molecular techniques and the fundamentals of biology are based on it. In 1973 E.M. Southern developed the Southern hybridization technique to detect DNA ^14^ and later in 1977, northern hybridization was developed by Alwine *et al*. to detect levels of RNA ^15^. In 1983, Dr Kary Mulis developed PCR (Polymerase Chain Reaction) for DNA amplification, which is also based on antiparallel complementary hybridization of DNA and for this he was awarded Nobel Prize in 1993 ^16^. In 1973, Boyer, Cohen and Chang developed cloning techniques which has allowed the production of recombinant protein and a whole new science of recombinant DNA technology has been developed based on this ^17^. On the basis of Watson and Crick model of DNA and using Sanger DNA sequencing chemistry, scientists throughout the world invested billions of dollars and developed the Human Genome Project ^18, 19^. Today efforts are being taken to sequence the genome of each organism. On the basis of homologous DNA sequence, gene knockout technology was developed and many scientists have tried to characterize functions of various proteins and DNA elements on the basis of gene knockouts ^20– 22^. Capecchi, Evans and Smithies, were awarded the Nobel Prize for developing gene knockout technology in 2007. In 1998, siRNA technology was developed by Andrew Z. Fire and Craig C. Mello for which they were awarded Nobel Prize in 2006 ^23^ and Microarray technology was developed by Pat Brown which provides powerful tools for global characterization of gene expression ^24^. Yes, truly whole molecular biology has flourished with much new information and technologies in last 60 years based on the Watson and Crick model of DNA and it will not be possible for me to write about all techniques.

## Villain of molecular biology

**Figure 1.**
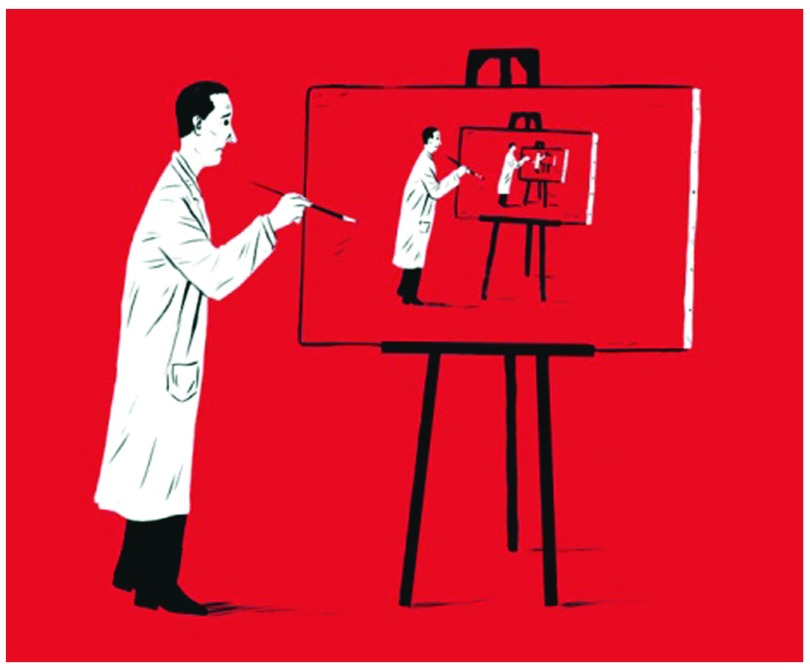
Image by Paul Blow in article “Reproducibility: The risks of replication drive” by Mina Bissel, *Nature*, 2013. Used with permission from Macmillan Publishers Ltd. *Nature* ©2013 ^54^.

There are many publications which have reported huge errors of various molecular techniques (for more details see 25– 36). You may have experienced, non-specific amplification of DNA in a PCR reaction, non-specific hybridization in Southern and northern hybridization and then have tried harder to find out conditions which give you better results. You may have experienced, non-specific cloning reactions and then must have tried to screen out a specific clone out of non-specific ones. Even today we do not have answer as to why petunia flowers turn white on overexpression of a gene which should have made it more purple ^37^. None of our gene knockout technology explains whether they have taken out one and only one gene and the remainder of the genes have not been affected. To the best of my knowledge, there is no whole genome sequence information available for knock out organisms. I wish to inform readers that the Human Genome Project is not yet complete even though its first draft was announced 15 years ago ^38^. All over the world, billions of dollars are still invested in a hope to find solutions for various diseases. How can we find solutions if the molecular techniques used show errors and many times we are unable to reproduce the same findings in different labs. In 2012 the Biotechnology Company Amgen with a team of 100 scientists found that only 10% (6 out of 53) of research published by reputable labs in top journals is reproducible and 90% of money ($28 billion) is wasted. It looks like that even after development of high throughput techniques and instruments, research worldwide is losing accuracy and precision. It is a worse situation for biotechnology/pharmaceutical industries who are going to invest or have invested millions of dollars for their new drug development programme. It’s again a far worse situation for the public who are looking forward to scientists one day finding solutions to deadly diseases and producing cheaper drugs and the best treatments soon ^39, 40^. Recently Professor Eric Lander (one of the leaders of the Human Genome Project, and a member of US President Barack Obama’s scientific advisory panel) visited India and gave an exclusive interview stating that we will have a solution for most cancers in the next 25–30 years ( http://www.ndtv.com/video/player/ndtv-special-ndtv-24x7/mapping-the-human-genome-the-eric-lander-interview/358410). I totally disagree with his statements as with the current ways of doing research, it may take many thousand years to find ultimate solutions for mankind’s problems. A recent report by John Arrowsmith revealed that the Phase II success rate for new development projects has decreased by 10% in the last few years. It will definitely increase the cost of new drugs in the future. It will also decrease the trust of the public Government and funding agencies in scientific activities ^41^. But still a question arises, who is the ‘villain’ behind these problems? Yes, the B- form of DNA is a ‘hero’ of molecular biology but there is also a ‘villain’ of molecular biology. It’s a form of DNA which is actually much less studied, discussed and used in designing molecular techniques. It is “parallel stranded duplex DNA” which was first reported by Ramsing & Jovin and Sande *et al.* in 1988. There are few reports in favour of parallel stranded DNA which summarize that there is no drastic difference in parallel and antiparallel DNA having mixed AT/GC composition ^42– 47^. Recently we have developed a PD-PCR technology based on parallel stranded DNA and we have concluded that two PCR products can be synthesized from a single stranded template DNA, one by conventional PCR and another by our approach ^48^. In 2008, Lestienne *et al.* reported a novel property of TFO (Triplex forming oligonucleotides-known for transcription inhibition) that Triple helix primer (THP) bounded to the duplex DNA in a parallel orientation can initiate DNA synthesis by various DNA polymerases of phage, retrovirus, bacteria and humans ^49, 50^. There are reports which state that Southern hybridization reaction can be performed using parallel complementary probe and gene silencing can be applied using parallel complementary RNA ^51– 53^. It also makes me think whether earlier scientists have developed a 100% accurate genome sequence of Human which has only been developed on the basis of antiparallel complementarity in DNA. I strongly believe technical errors observed in various molecular techniques can be ruled out by considering both parallel and antiparallel complementarity of DNA. A probe for Southern blotting/northern blotting can be designed such that it binds to its target only in an antiparallel manner. Primers for PCR can be designed in a similar way. There is a need to develop siRNA and microarray chips keeping in mind parallel and antiparallel hybridization of DNA. Science without errors will increase reproducibility in research worldwide ( Figure 1).
