# Supplementary material for: Villain of Molecular Biology: Why are we not reproducible in research?
Source: F1000Res. 2015 Jul 30;4:438. [Version 1] doi: 10.12688/f1000research.6854.1 (PMC4544406; doi:10.12688/f1000research.6854.1)
Supplement: Peer review history

# Peer review: Referee response for version 1

**DOI:** 10.5256/f1000research.7374.r9738

**Published:** 2015-08-10

**Authors:** Nirajkumar Singh

**Affiliations:** 1 Department of Biotechnology, Shri A. N. Patel PG Institute, Anand, India

The article presented here “Villain of Molecular Biology: Why are we not reproducible in research?” deals mainly with the issue of non-reproducibility of research citing many examples including the example of DNA structure elucidated by Watson and Crick.

I feel this is very serious issue throughout the world. The author claims only about 10% research paper work is reproducible. However, keeping the recent development of genetic engineering and its product (used in day to day life e.g., Insulin, BT Cotton, and many more..) in mind, I think this figure should be more than that mentioned by authors, even though the authors are very much correct about the reproducibility of scientific work.

Overall the manuscript is written in a very systematic way which fully justifies the title of the manuscript. In my opinion the manuscript is adequate for indexing.

I have read this submission. I believe that I have an appropriate level of expertise to confirm that it is of an acceptable scientific standard.

# Peer review: Referee response for version 1

**DOI:** 10.5256/f1000research.7374.r9734

**Published:** 2015-08-03

**Authors:** Sukrat Sinha

**Affiliations:** 1 Emory Vaccine Center, Emory University, Atlanta, GA, USA

The article written by Dr. Vikas Bharadwaj throws light on the issue of non reproducibility of research citing the example of DNA structure elucidated by Watson and Crick. Every year billions of dollars are being spent on this part of research. The article envisages the very valid point that there is a dire need to revise the concepts so that our research could be more fruitful and result oriented. I highly appreciate Dr. Bhardwaj for bringing this issue on forefront.

I have read this submission. I believe that I have an appropriate level of expertise to confirm that it is of an acceptable scientific standard.
